# Varietal diversity and conservation status of banana, taro, pumpkin, and mustard green in mountainous areas of Northwest Vietnam

**DOI:** 10.1007/s13280-025-02262-1

**Published:** 2025-10-23

**Authors:** Dang Toan Vu, Phuong Diep Vien Ta, Tuong Dang Vu, Diego Naziri, Thi Minh Thao Le, Anh Thu Vo, Israel Navarrete, Stef de Haan

**Affiliations:** 1 Plant Resources Center, Vietnam Academy of Agricultural Sciences, An Khanh, Hoai Duc, Hanoi, Vietnam; 2 Department of Agriculture and Forestry, Thai Nguyen University, 13 Cluster, Binh Minh Ward, Lao Cai, Vietnam; 3 School of Agriculture and Food Sustainability, University of Queensland, 5391 Warrego Hwy, Gatton, QLD 4343 Australia; 4 Earth and Life Institute, Université Catholique de Louvain, Louvain-la-Neuve, Belgium; 5 International Potato Center, Km 2 Pham Van Dong, Hanoi, Vietnam; 6 Natural Resources Institute (NRI), University of Greenwich, Chatham Maritime, UK; 7 International Potato Center, Panamericana Sur Km 1, Quito, Ecuador; 8 International Potato Center, Avenida La Molina 1895, Lima, Peru; 9 Biosystematics Group, Wageningen University and Research, Wageningen, The Netherlands

**Keywords:** 5-cell analysis, Agrobiodiversity, Ethnobotanical uses, Vietnam

## Abstract

This study assesses the agrobiodiversity richness, traditional knowledge, and conservation status associated with four key crops—banana, taro, pumpkin, and mustard green—in Northwest Vietnam. Using the 5-cell method, 133 farmer varieties were identified in Mai Son and Sa Pa districts. Mai Son showed the highest level of varietal diversity in banana and pumpkin, while Sa Pa had the higher richness in mustard green and taro. Ethnic groups, particularly Thai and Dao households, played a key role in maintaining this diversity. The findings reveal significant vulnerability to varietal loss, particularly for banana and taro. In contrast, pumpkin varieties in Sa Pa and mustard greens in both districts appear less vulnerable. Declining cultivation of certain farmer varieties is driven by changing preferences, market limitations, and climate variability. Results underscore the need for on-farm conservation strategies such as custodian farmer networks, biodiversity seed fairs, seed banks, improved market access, and nutritional education, to safeguard local varietal diversity and promote sustainable farming systems in the region.

**Supplementary Information:**

The online version contains supplementary material available at 10.1007/s13280-025-02262-1.

## Introduction

Agrobiodiversity encompasses the variety and variability of living organisms that are used directly or indirectly for food and agriculture. This includes crops, livestock, and microorganisms that are essential for food production and ecosystem functions (FAO 1999). The diversity of species, farmer varieties, and animal breeds is the result of combined natural selection processes and the careful selection of farmers, herders, and fishers over millennia (FAO 2004). At crop level, diversity consists of the range of species managed by farmers (interspecific variability) and the diversity of farmer varieties—often referred to as landraces, traditional, indigenous or ancestral varieties—within each species (intraspecific variability). These are the result of the continuous heterogeneous interaction between socioecological conditions such as ethnobotanical, agroecological and food system rationales of farmers, and biophysical conditions such as biotic and abiotic selection factors (Zimmerer and de Haan 2019).

It is widely recognized that indigenous people—referred to as ethnic minority groups in most of Southeast Asia—play a crucial role in managing agrobiodiversity through their traditional knowledge, traditional farming practices, and biocultural attachment to crop diversity as expressed in cuisine and cosmovision (Pilgrim and Pretty 2010; Agnoletti and Santoro 2022; Ba et al. 2023). Agrobiodiversity is crucial to global food and nutrition security, climate adaptation, and smallholder resilience, especially in the most marginal and vulnerable environments (Martins 2015; Zimmerer et al. 2019). How this biodiversity is nurtured and managed determines the security of future food supplies and the sustainability of productive ecosystems (Vandermeer et al. 2002). Indigenous people in centers of crop and livestock origin and diversity provide an important ecosystem service sustaining the process of ongoing evolution of genetic resources and associated traditional knowledge. This key contribution is recognized in multiple international treaties, including the Nagoya Protocol and Kunming–Montreal Global Biodiversity Framework under the Convention on Biological Diversity (CBD) and Article 9 of the International Treaty on Plant Genetic Resources for Food and Agriculture (ITPGRFA). However, there can be important differences between different ethnic groups sharing common geographies when it comes to varietal diversity, conservation practices, and associated knowledge (e.g., Perales et al. 2005; Rana et al. 2007). Such differences have been explained by specific agroecological, social, and cultural factors (e.g., Labeyrie et al. 2014; Westengen et al. 2014).

Globally, the ongoing on-farm conservation of cultivated species and varietal diversity is reported to be increasingly threatened by natural and anthropogenic drivers (FAO 2025). These drivers consist of climate-induced variability and extreme weather events, land conversion affecting crop-wild relative interactions, agricultural intensification and specialization, as well as changing global food supply chains and diets (Bijlsma and Loeschcke 2011; Khoury et al 2014). How rural households respond to rapid socioeconomic and demographic changes inevitably involves trade-offs for indigenous crops and varietal portfolios (Chaudhary et al. 2020). Yet, our scientific understanding of genetic erosion remains largely nonsystematic in the sense that research on the phenomenon of varietal change is generally characterized by a lack of baselines and consistent timeline studies. Many specific studies on genetic erosion simultaneously report the occurrence of new or previously unreported diversity (Khoury et al. 2021). Displacement and replacement rates of farmer varieties vary considerably between countries, regions and crops (Van de Wouw et al. 2009; Gatto et al. 2021). However, mountain regions are commonly considered modern-day “refugia” of crop genetic diversity because of their agroecological and environmental complexity, cultural richness, isolation, marginality, and imperfect integration into mainstream market systems (Brush 2004).

The global and fine-grained hotspot-level spatiotemporal dynamics of crop diversity remains poorly understood. Importantly, for key centers of crop origin and diversity this knowledge gap challenges the design of appropriate conservation strategies (Dulloo et al. 2017). Conservation monitoring concerning so-called neglected, underutilized or orphan crop species, and their farmer varieties is particularly limited (Padulosi et al. 2012). An initial step to understand the conservation dynamics and ascertain if and how crop genetic diversity is either preserved, lost or enriched, is to assess its current status. This information can in turn be used to establish baselines for setting up long-term monitoring systems which would allow to assess the change of species and farmer varietal portfolio over space and time, improve the design of in situ and ex situ conservation or restoration programs, and strengthen policies and regulations for effective conservation (de Carvalho et al. 2016). A series of monitoring frameworks and specific tools and methods have been developed (de Haan et al. 2016; Dulloo et al. 2021; Almeida et al. 2024), yet their implementation requires concerted action, common procedures, and long-term commitment and finance.

Northwestern Vietnam is representative of the scientific challenges outlined above. The country is geographically located in Indochina, one of the world’s main centers of biodiversity (Myers et al. 2000). It is globally known as one of the most important biodiversity hotspots (Vu et al. 2022), and part of the center of origin and diversity of essential crops such as rice (*Oryza sativa* L.*),* taro (*Colacasia esculenta* (L.) Schott), banana (*Musa* L.), and a wide range of indigenous vegetables (Siemonsma and Piluek 1993; Trinh 1996; Le Dinh Danh et al. 1998; Vu et al. 2023). Vietnam is also an ethnically diverse country with 54 officially recognized ethnic groups, including the Kinh (Vietnamese) majority, and high levels of ethnolinguistic diversity concentrated in the northern mountainous areas. Groups often differ by crop portfolio, farming practices, food uses and traditions, even when inhabiting the same geography (Vu et al. 2024a). These ethnic minority groups produce a large diversity of crops, which are used for home consumption and sale, contributing to nutrition security and income generation (Ky et al. 2003). While there is some evidence that the number of traditional crop species managed by ethnic minority households has decreased, there is an overall lack of species specific comparative studies among different ethnic minority groups. Diversity loss has been commonly attributed to a progressive shift toward monoculture practices and the adoption of modern high-yielding varieties of the most commercial crops such as rice, cassava, and maize (Thuaire et al. 2021). This may directly affect the resilience of production systems and the livelihoods of local communities (MONRE 2014).

There is insufficient systematic evidence on the conservation status of key crop species and their farmer varietal diversity in Vietnam, particularly for crops originating in, or having key recognized hotspots within, the country. But also concerning differences among different ethnic groups and the associated knowledge. Despite the recognized importance of crop diversity, there is limited systematic, comparable information on (i) the current varietal diversity of key indigenous crops in representative crop and ethnolinguistic hotspots of Vietnam, (ii) the factors driving retention, loss, or enrichment of varieties (socioeconomic, ecological, cultural drivers), and (iii) the conservation status of farmer varieties (extent of current on-farm cultivation). Without this evidence, conservation priorities or interventions cannot be targeted effectively, risking the loss of unique genetic resources and the ecosystem and livelihood services they support.

In this study, we aim to partially address the challenge and knowledge gap outlined above by assessing the conservation status of intraspecific diversity for four globally and locally important crops, for which the country overlaps with potential centers of origin and diversity: banana (*Musa* sp*.*), taro and other edible aroids hereinafter collectively referred to as taro (Araceae family), pumpkin (*Cucurbita* sp.), and mustard green (*Brassica* sp.). Furthermore, we examine the main trends in agrobiodiversity dynamics such as increase or decline of specific farmer varieties, the underlying drivers, and document the traditional ethnobotanical knowledge and use associated with farmer varieties. We aim to address the following research questions: (i) What is the varietal richness of these four crops managed in different locations of northwestern Vietnam by different ethnic groups? (ii) What is the ethnobotanical knowledge associated with the farmer varieties of these crops? (iii) What is the conservation status of these varieties and what are the main drivers of change? (iv) What are the main trends in cultivation of the farmer varieties? The study sites were selected for their exceptionally high levels of intraspecific diversity and their diverse ethnic makeup, which offers an excellent basis for comparison. The findings can inform strategies for promoting the conservation and use of agrobiodiversity in the mountainous areas of northern Vietnam (Government of Vietnam 2022), thus contributing to the much-needed transition toward a more sustainable agricultural system as outlined in the National Action Plan for Food System Transformation and in the National Agrobiodiversity Strategy (Government of Vietnam 2023).

## Materials and methods

### Study area

**Fig. 1:**
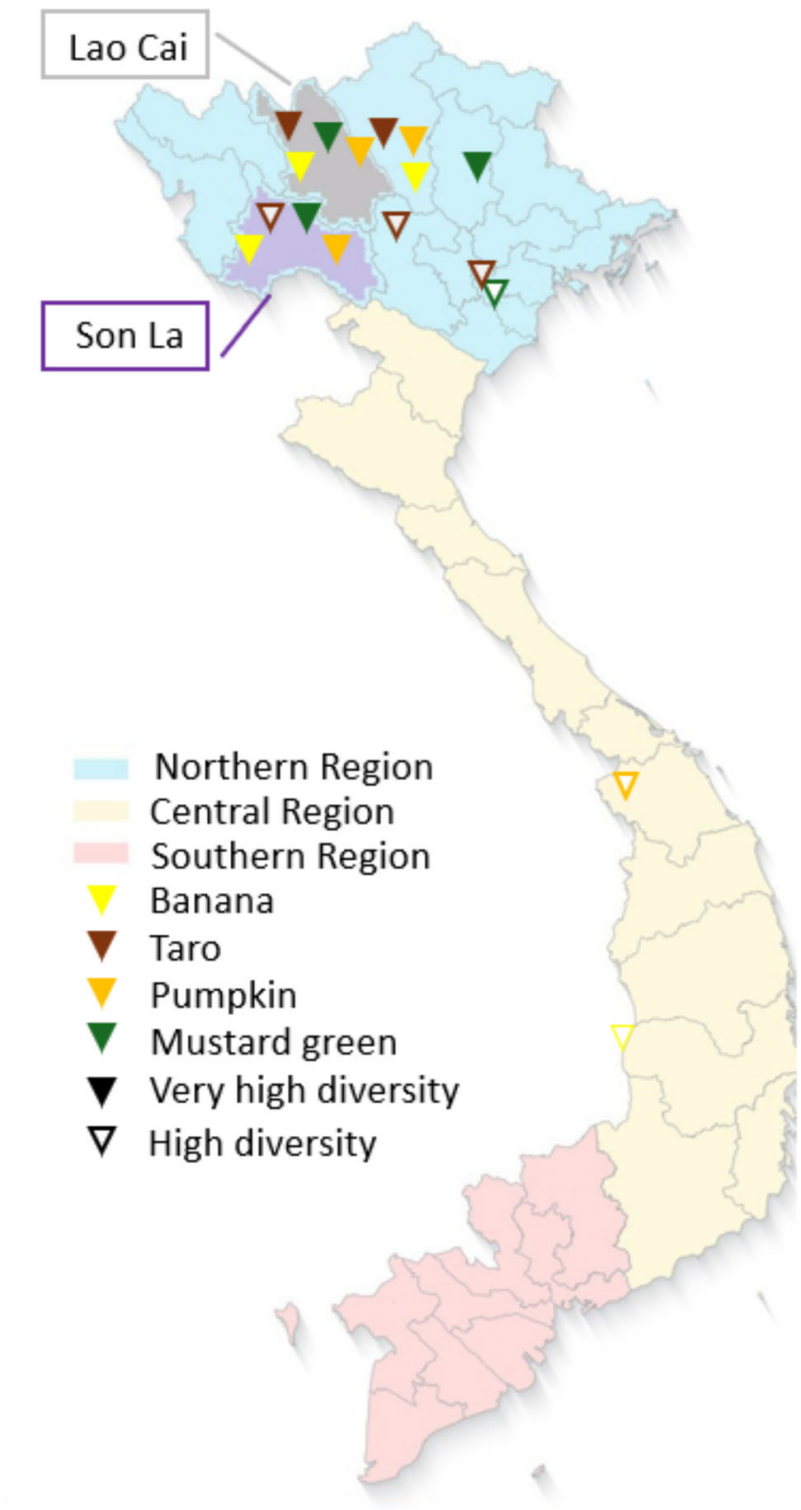
Provinces of Vietnam hosting the highest intraspecific diversity for banana, taro, pumpkin, and mustard green based on Plant Resources Center expert consultation

The study focused on two provinces of the northwest mountainous region of Vietnam: Son La and Lao Cai. These provinces are assumed to host some of the highest levels of intraspecific diversity in the country for the four target crops (Fig. 1; Nabuuma et al. 2021; Hoang et al. 2023; Vu et al. 2023). Within these provinces, the study was conducted in districts of Mai Son and Sa Pa.

Mai Son is located at the center of Son La province, covering an area of 1410 km^2^ at elevations of 800–1500 m. The 2023 population was estimated at 169 000 and consisted of six major ethnic groups: Thai, Kinh, Hmong, Xinh Mun, Kho Mu, and Muong. This district has a tropical monsoon climate with an average temperature of 21 °C and an average annual rainfall of 1415 mm. The agricultural production area is estimated at 60 555 Ha, with extensive cultivation of longan, sugarcane, mango, strawberries, and coffee, alongside rice and maize (Son La PSO 2023). In this district, most agricultural production is meant for sale, with fruits and vegetables being especially important for income generation, besides food security (Boukaka et al. 2024).

Sa Pa is a highland district located in the western part of Lao Cai province, spanning about 680 km^2^ at an altitude of 1500–1800 m. The 2023 population was estimated at 72 000, belonging to different ethnic groups such as Hmong, Dao, Tay, Giay, Xa Pho, Kinh, and Hoa. Sa Pa has a subtropical highland climate, characterized by mild winters and cool summers (average annual temperature is 15°–18 °C), and abundant rainfall (average of 2440 mm), especially during the monsoon period from May to September. Compared to Mai Son, the average land holding is smaller (0.4 vs. 1.1 Ha), farming is primarily for household consumption, and production is less diversified (Boukaka et al. 2024). Rice and maize occupy the majority of the estimated 9598 Ha of agricultural production area (Lao Cai PSO 2023). However, fruits and vegetables are important crops for food security and, often, as a source of income too when surplus production is sold to local markets. Sa Pa is also a major tourist destination in Vietnam which provides opportunities for income diversification.

Mai Son and Sa Pa districts were identified as strategic locations to conduct the study for different reasons. First, within the selected provinces, these districts host an abundance of varieties of all four target crops (Vu et al. 2024b, c). Second, the traditional plant genetic resources have been reported at risk of erosion, as people in these places are changing their farming methods and increasingly switching to modern varieties (Ky et al. 2003). Third, the ethnic makeup between the two districts is considerably different, providing a valuable context for contrasting patterns and practices. The study was conducted in eight (8) different villages in four (4) priority communes of Mai Son and Sa Pa districts. Table 1 lists the study villages and presents some basic demographic characteristics while maps of the study sites are provided in Appendix S1 in Supplementary Information. The communes and villages were identified in consultation with the Plant Resources Center (national genebank) and local authorities as traditionally possessing a high level of agrobiodiversity, particularly for the four target crops.

**Table 1:** List of target villages and main ethnic groups of residents. *Source* adapted from Loc (2022)

District	Commune	Village	Population	Main ethnicity
Sa Pa	Muong Hoa	Thao Hong Den	885	Hmong
		Hoa Su Pan 1	927	Hmong
	Thanh Binh	Lech Dao	779	Dao
		Kim	760	Dao
Mai Son	Co Noi	Tan Thao	235	Kinh
		Mon	987	Thai
	Chieng Luong	Y Luong	847	Thai
		Mon 2	758	Thai

### Data collection and analysis

**Fig. 2:**
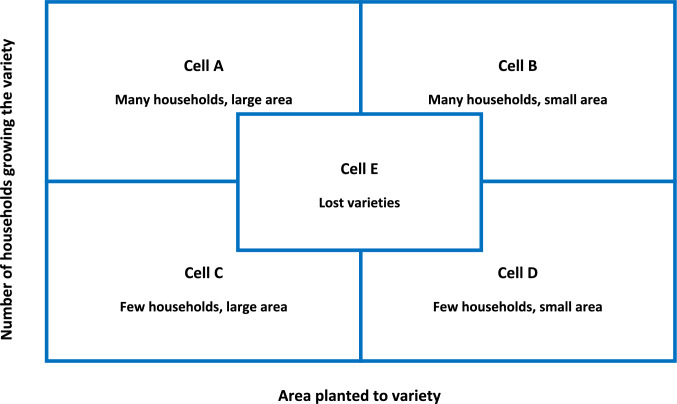
Graphic representation of the 5-cell analysis (*adapted from *Dulloo et al. 2017)

The agrobiodiversity baseline assessment was conducted using the 5-cell analysis, a participatory method to assess the amount and distribution of local crop diversity within farming communities (Dulloo et al. 2017, 2021). Its objectives are twofold. First, to identify common, unique, and rare crop farmer varieties in a given location by categorizing them into groups of varieties that occupy large or small areas and those farmer varieties that are grown by many and few households (Fig. 2). The technique also allows identifying varieties that have already been lost (OXFAM 2018). Second, to document the main perceived reasons why the varieties are maintained or being lost.

**Fig. 3:**
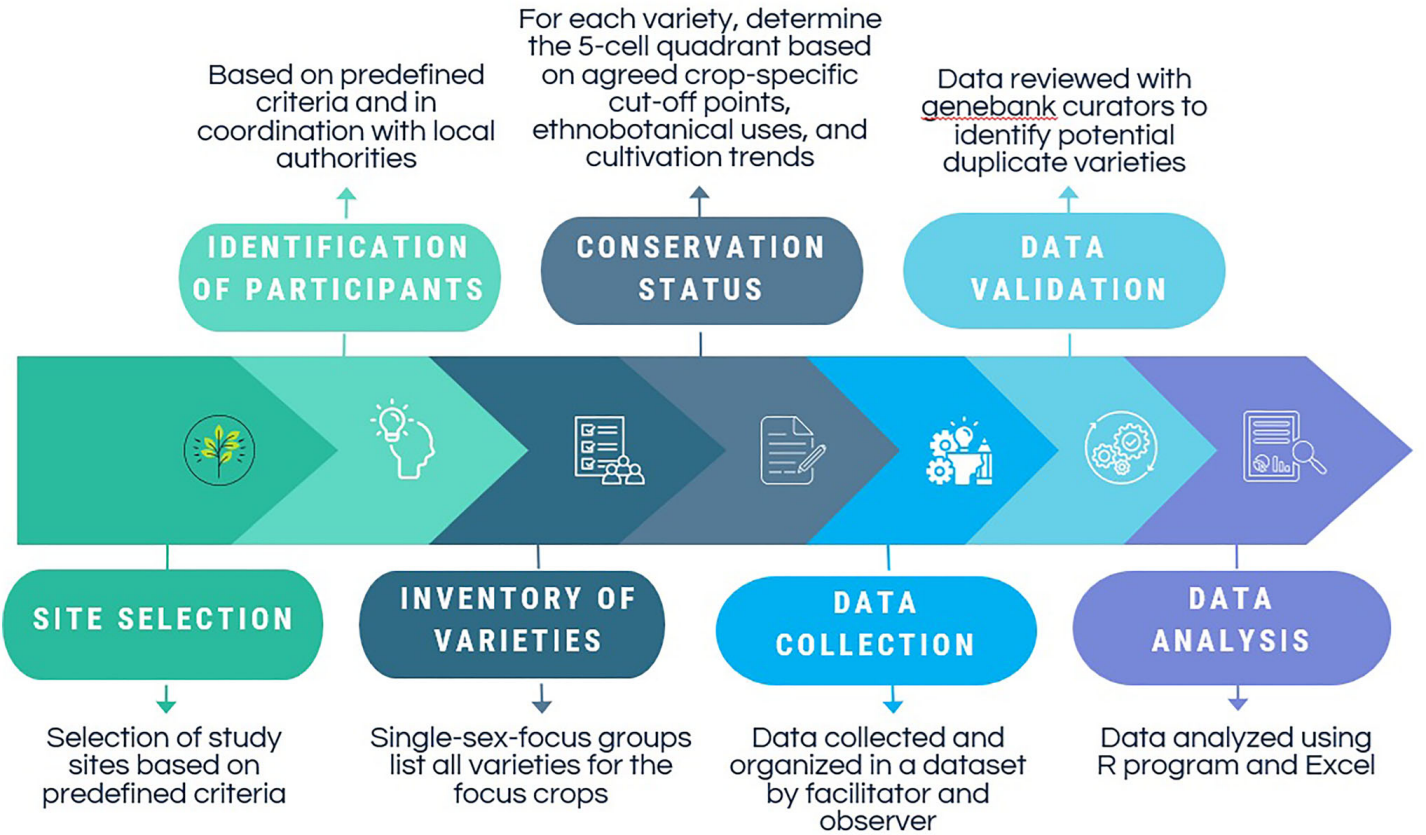
Methodological diagram

In order to deploy the 5-cell method, a workshop was organized in each of the eight villages in July 2023. The steps followed are illustrated in the methodological diagram (Fig. 3). Participants were identified in coordination with the local authorities, namely the Department of Agriculture and Rural Development (DARD) in Mai Son and the District People Committee in Sa Pa. Criteria for selecting the participants included being actively engaged in cultivation and having extensive knowledge of the four target crops, while also ensuring, as much as possible, balanced gender representation. A total of 107 participants (61 females and 46 males) joined the workshops, ranging from 10 to 17 per village. In each workshop, participants were divided in two sex-disaggregated focus groups. For each crop, they were initially tasked to list the varieties they grow or are aware of in their locality. In order to ease the assignment and the discussion among participants, whenever possible, they were asked to bring samples of each local farmer variety. Once the listing exercise was completed, a long unique list was compiled, comprising all varieties of the crop that had been reported in the separate focus groups. All men and women participants were then brought together and, after familiarizing themselves with the consolidated list, invited to discuss and agree on the most sensible cutoff points for defining whether a variety of that crop was grown in small or large areas, and by few or many households. Following the definition of these cutoff points,^1^ the participants discussed and agreed on the cell (quadrant) that best represented the current conservation status of each farmer variety. Each variety was then written on a sticky card which was placed in the agreed quadrant on a large diagram drawn on a flip chart (similar to the one shown in Fig. 2). For each reported variety, the ethnobotanical uses and the trends in cultivation over the past 10 years were also enquired (the latter categorized as increase, decrease, unchanged, or unknown; and, where a change was reported, the main reason for it) using a semi-structured questionnaire and recorded in a notebook. The same process was then repeated for the other three crops. A facilitator and an observer were assigned to each workshop. The facilitator was in charge of asking questions, encouraging a lively atmosphere, and ensuring that all participants actively contributed to the discussion. The observer was in charge to take and organize the notes. In all cases, except with the Kinh ethnic group in Mai Son, support was provided by local authorities for interpretation from local languages to Vietnamese.

The collected data were cleaned and discussed with staff of the national genebank to identify duplicate varieties that could be referred to with different names by different ethnic groups within the same district. The data were then organized in a structured dataset (Naziri et al. 2024) and analyzed using R (version 4.3.3) and Microsoft Excel. Descriptive statistical methods were applied, with results presented as frequencies, proportions, and mean values.

## Results

### Varietal diversity managed by farming communities

**Fig. 4:**
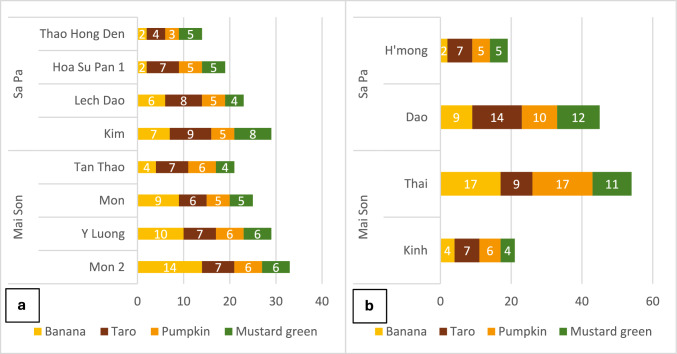
Number of varieties reported by crop and village (**a**) and by crop and ethnic group (**b**)

A total of 133 varieties were reported by the study participants: 69 in Mai Son and 64 in Sa Pa. Some examples are shown in Appendix S2 in Supplementary Information. Across the two districts, the highest number of farmer varieties was recorded for pumpkin (*n* = 38), followed by taro (*n* = 34), mustard green (*n* = 32), and banana (*n* = 29). The highest number of varieties for pumpkin and banana was found in Mai Son (23 vs. 15 and 18 vs. 11, respectively), while for taro and mustard green in Sa Pa (21 vs. 13 and 17 vs. 15, respectively; see Appendix S3 in Supplementary Information). At village level, Mon 2 village in Mai Son reported the highest diversity of varieties for the four target crops (33 varieties), followed by Kim village in Sa Pa and Y Luong village in Mai Son (29 varieties each), and Mon village in Mai Son (25 varieties) (Fig. 4). Furthermore, Mon 2 village had the highest number of bananas, with 14 distinct varieties. For taro and mustard, the highest varietal diversity was found in Kim village (nine and eight farmer varieties, respectively). In the case of pumpkin, three villages in Mai Son showed the highest diversity, reporting six varieties each.

Overall, when disaggregated by ethnic group, Thai people in Mai Son manage the highest number of varieties, especially of banana and pumpkin. For all crops, the Thai maintain more diversity than the Kinh who inhabit the same district. Similarly, the Dao in Sa Pa manage the highest diversity for taro and mustard and, for all crops, maintain more varieties than the Hmong in the same district (Fig. 4).

### Ethnobotanical uses

Focus group participants indicated that most varieties (76%) have multiple uses, often associated with a specific part of the plant. Noticeably, the banana variety *Cò mặc cuổi kéo*, grown by the Thai’s community, exhibited nine distinct patterns of usage. With regard to taro, the variety *Phác sọ óoc*, also managed by the Thai people, was notable for having the highest number (4) of reported usages. The pumpkin varieties *Phản nhum chuỳ*, *Phản nhum đao*, *Phản nhum chùn*, *Phản nhum bụt*, *Phản nhum pìn* grown by the Dao ethnic group are also used in four different ways. The most common usage of these crops is as food (over 93% of varieties), followed by fodder (53%), sale (50%), medicine (15%) and other uses (5%). This suggests that the ability to address the three basic needs—food, fodder, and additional income through sales—are critical factors influencing farmers’ decisions to maintain and continue cultivating these farmer varieties.

The part of the plants which are used also vary across the crops. For bananas, pseudostem or “trunk” was the most frequently utilized part (86% of varieties), mostly as fodder, followed by fruit (79%), for both culinary and sale purposes, flower (41%), leaf (24%), and the whole plant (7%) as seedling.^2^ With regard to taro, corm and petiole were the most commonly used part (71%), either for household consumption or sale, followed by leaf (62%), mostly as fodder. For pumpkins, the fruit was used in 88% of the reported farmer varieties, followed by young shoots (72%) and leaves (56%). In the case of mustard greens, for all varieties, the whole plant, except the roots, was used, mostly for household consumption or sale.

In terms of preparation methods, the crops were predominantly used after being cooked, which accounted for 66% of banana varieties and all farmer varieties of taro, pumpkin, and green mustard. For all crops, except mustard green, some specific varieties are used for traditional medicine. Some notable preparation methods for treating illnesses include decoction, sugar soaking, alcohol soaking, or milling.

For each of the reported varieties, the name meaning, and how the different parts of the plants are prepared and used by the local communities are presented in Appendix S4 in Supplementary Information.

### Current conservation status

**Fig. 5:**
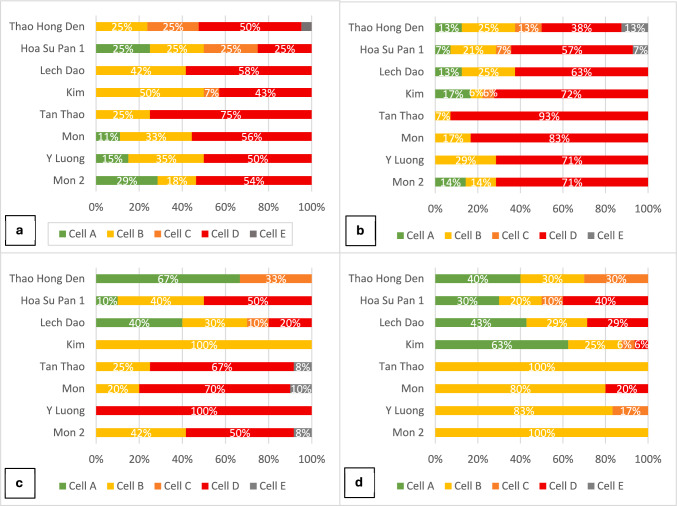
Share of varieties in the 5-cell quadrants by village with respect to a: banana, b: taro, c: pumpkin, d: mustard green *(Cell A* = *Many households and large areas; Cell B* = *Many households and small areas; Cell C* = *Few households and large areas; Cell D* = *Few households and small areas; Cell E* = *Lost varieties)*

Figure 5 shows the relative abundance (i.e., amount and distribution of diversity) of the reported farmer varieties in each village, based on the classification discussed and agreed upon by the participants in the workshops.

Our results indicate that the diversity of bananas and taros in all study villages of both districts, and pumpkins in Mai Son district is under threat. Indeed, with the exception of only two villages in Sa Pa for banana (Hoa Su Pan 1 and Kim), there was a consensus among focus groups participants that at least half of the farmer varieties of the crops studied in these locations (and up to 100% in the case of Y Luong village for pumpkin) are cultivated primarily by a few households on small areas, or have already been lost. Such a high proportion of varieties being cultivated by few households on small areas indicate that they are rare and at high risk of extinction—with varietal loss already being reported for some varieties of taro in two villages of Sa Pa and for pumpkin in three villages of Mai Son. In all these cases, with the only exception of pumpkin in one village in Mai Son (Y Luong) and taro in one village in Sa Pa (Kim), the second most frequent categorization refers to varieties being cultivated by many households on a small areas, which usually indicates that they are culturally important varieties, distributed quite uniformly in the landscape, and important for food security.

Also, for mustard green in Mai Son, the majority of varieties in all villages are cultivated by many households on a small area. In this case, the conservation status is less concerning as very few varieties are cultivated by few farmers on a small area (in one village only, Mon, where 20% of farmer varieties fall in this category). Finally, the conservation status of pumpkin and mustard green in the villages of Sa Pa district is the least concerning as most varieties are cultivated by many farmers, either on large or small areas.

It is noticeable that, unlike for taro and banana, the conservation status of pumpkin and mustard green is noticeably different between the two districts, with Mai Son being more a source of concern, especially for pumpkins for which, indeed, about 10% of varieties were reported as being already lost. This, together with one taro variety in Sa Pa, were the only cases where farmers indicated that loss of varieties had already occurred.

An additional analysis was conducted to identify the varieties at the highest risk of loss in the two districts. This analysis aimed to address cases where a variety was reported as threatened or already lost in one village but was still widely cultivated in another village within the same district, indicating that it might not actually be at high risk of loss. The varieties that have been reported by all focus groups conducted in a district as being cultivated by few households on a small area, or already being lost are presented in Appendix S5 in Supplementary Information (additional details are provided in Appendix S6). The results confirm the comparatively high risk of varietal loss for banana and taro’s varieties across both districts, and of pumpkins in Mai Son only.

### Trends in cultivation and associated drivers

**Fig. 6:**
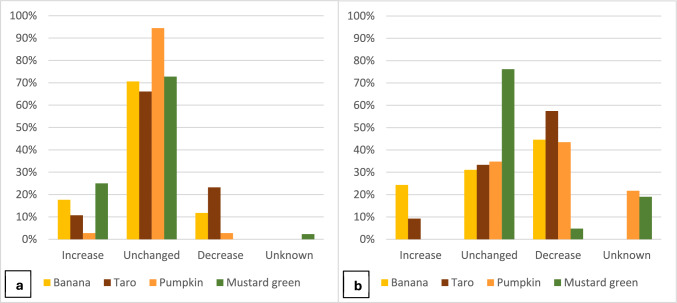
Share of varieties of target crops for which the cultivation area has increased, decreased, or remained unchanged over the last 10 years with respect to **a**: Sa Pa district, **b**: Mai Son district

The trend in the cultivation of the identified varieties over the last 10 years was found noticeably different between the two target districts. In Sa Pa, over 70% of the farmer varieties of all four target crops have not undergone significant change in allocated area during this period. This is especially true for pumpkin with 94% of the varieties having experienced no significant change in cultivation area (Fig. 6). Mustard green’s varieties witnessed the highest increase in cultivation areas, which was reported for over 30% of farmer varieties. This can be explained by the strong representation of the Hmong ethnic community in Sa Pa which is associated with the traditional cultivation and uses of these mustards that are increasingly sold to the many restaurants, home stays, and other tourist outlets. In the case of banana and taro, the results indicate that about 10–20% of the farmer varieties have experienced either an increase or a decrease in cultivated areas, suggesting a possible ongoing displacement of several farmer varieties with other ones, possibly with high yields or market demand.

In the case of Mai Son, the findings are quite different. In this district, no major shifts were found only for mustard green, while the cultivated area of over 55% of the farmer varieties of taro and 40% of varieties of banana and pumpkin has decreased over the last 10 years. For banana, and lesser extent taro, the reduction in cultivated area for many farmer varieties is partially offset by the increase in areas allocated to other varieties. This is similar to the case of Sa Pa for the same crops, suggesting an ongoing partial displacement and turnover of varieties, which can signal a threat to certain farmer varieties but also highlight the dynamic nature of in situ conservation. With regard to pumpkin, the area allocated to individual farmer varieties has either remained unchanged or decreased, also pointing out at a possible risk of loss of varietal diversity.

Participants were asked about the main reasons for reducing or abandoning the cultivation of a given variety. The primary reason was the lack of farmer’s interest which was associated with insufficient market demand, limited market access, and profitability, or the loss of cultural attachment and valuing specific varieties as part of a key component in local cuisine. Other notable factors were related to preferences for other crops and varieties, and disadvantages in cultivation, including susceptibility to pests and diseases, and vulnerability to climate change. These factors were reported especially for banana and taro varieties, and, to a lesser extent, for pumpkins, while their impact seems negligible for varieties of mustard green. These drivers and their relevance for the varietal diversity of each crop underpins the results concerning their conservation status.

## Discussion

Agrobiodiversity is a foundational pillar of sustainable development as it underpins food systems, cultural identity, rural economies, climate adaptation, and ecosystem health. Without it, achieving the Sustainable Development Goals (SDGs) becomes harder because we risk creating fragile food systems and reducing resilience to global change. The conservation of agrobiodiversity and varietal diversity is particularly relevant to poverty reduction and livelihoods (SDG 1), food security and nutrition (SDG 2), climate resilience and environmental sustainability (SDGs 13 and 15), sustainable consumption and production (SDG 12), cultural heritage and indigenous knowledge (SDGs 11 and 4), and health and well-being (SDG 3).

This study reports on the rich varietal diversity and conservation status of banana, taro, pumpkin, and mustard green across four ethnic groups in two mountainous districts of Northwest Vietnam, namely Sa Pa and Mai Son. These groups, including Kinh, Thai, Hmong, and Dao, play a pivotal role in maintaining varietal diversity. Overall, the local farmer communities studied manage over 130 nominal varieties, which were fairly evenly distributed among the four crops (29–38 farmer varieties per crop). Mai Son showed higher levels of varietal richness for banana and pumpkin (18 and 23 varieties, respectively), and Sa Pa for taro and mustard green (21 and 17 varieties, respectively). These findings confirm that specific geography affects the detailed patterning of the varietal diversity of different crops which in turn can be influenced by specific landscape diversity, climatic, food culture, connectivity, and/or market factors (Zimmerer and Vanek 2016; Chiaffarelli and Vagge 2025).

The Thai community in Mai Son manages the highest levels of varietal diversity (54 varieties total), particularly for banana (17 varieties) and pumpkin (17 varieties), leveraging traditional knowledge and cultural practices related to these portfolios. Similarly, the Dao community in Sa Pa manages high levels of varietal diversity (45 total), especially for taro (14 varieties) and mustard green (12 varieties). Levels of varietal richness were substantially lower for the Kinh and Hmong ethnic groups with a total of 21 and 19 varieties for the four crops researched. This confirms results from previous studies highlighting the high levels of diversity managed by the Thai ethnic groups and comparatively lower levels of diversity by Hmong people among other ethnic minority groups inhabiting the same geographical area (Ha Dinh et al. 2003; Nguyen et al. 2023). This can in part be explained by the agroecological zones in which different ethnic groups reside with the Thai communities living in fertile valleys and the Hmong communities in the rain-dependent uplands. Yet, other factors, such as cultural and knowledge systems (e.g., food culture, cosmovision), socioeconomic reality (market integration, access to resources), political and institutional setting (policy, projects), historical and demographic issues (migration, intermarriage), and social organization and gender roles (e.g., collective vs. individual decision-making), can also have a significant role (Labeyrie et al. 2014; Shen et al. 2017).

From an ethnobotanical perspective, we noted the different characteristics and preferences that the local communities associate to each variety. Our findings also highlight the various factors influencing the decision to cultivate a particular variety, and how different parts of the plants are traditionally prepared and utilized for diverse purposes, spanning from food, animal feed to medicinal use. The use of farmer varieties in traditional medicine has been documented in several studies. For instance, Cahyanto et al. (2020) reported that in Indonesia banana sap of certain varieties was used to prevent infection, stick the wounds, stop bleeding, and dry the wounds, although these practices were falling into disuse. It is also well documented that different parts of taro and banana plants are used in different food cultures (Padam et al. 2014; Ferdaus et al. 2023). Clearly, multiple and multipurpose uses of farmer varieties are arguably an important driver of conservation in Vietnam’s northwestern highlands.

The 5-cell analysis revealed significant geographical disparities in varietal richness and threats to varietal diversity. Banana and taro showed potential trends of decline in both Sa Pa and Mai Son, with most farmer varieties being cultivated by few households on small areas. While this may signal their vulnerability to loss or genetic erosion, this may also represent a pattern of how certain groups of varieties are simply less prevalent. Nevertheless, nine banana varieties out of 29 (mostly in Mai Son) and 15 taro varieties out of 34 (mostly in Sa Pa) can be considered endangered. Pumpkin, with the highest varietal count, displayed a relatively robust conservation status in Sa Pa, where most varieties are cultivated on larger areas or by many households. In this district, no pumpkin variety was found endangered. Conversely, in Mai Son, almost half of the identified farmer varieties of pumpkin (10 out of 23) were found to be at risk with a vulnerable conservation status. Overall, in both districts, farmer varieties of mustard green are at low risk of loss, and no variety was found to be threatened.

Our findings also show that the cultivation of several banana and taro varieties has reduced in both districts over the last decade. The results suggest that this is likely due to a process of progressive substitution of local farmer varieties with exotic ones. This dynamic is stronger in Mai Son, possibly related to its farming system being more commercially oriented, with far better market linkages than Sa Pa where agriculture is still primarily a subsistence activity for home consumption. Also, for pumpkin in Mai Son, over 40% of the farmer varieties have experienced a reduction in the extent of cultivation. However, in this case no exotic varieties with increased areal distribution were found, suggesting a narrowing of the genetic diversity within the existing varietal portfolio.

Market access, changing preferences, and climate variability emerged as critical drivers influencing the conservation status. For instance, mustard green varieties were not reported at threat in Sa Pa. This is likely due to the recent expansion of the cultivation areas, in turn driven partially by the crop’s economic value and its growing use for food in the tourism service sector. Yet, the cultural importance and food preferences within the Hmong ethnic group remain also critical. This is also in line with other studies which indicate that, in terms of anthropogenic drivers, the change in farmer’s food preferences is one of the key causes influencing the conservation dynamics of varietal diversity (Chaudhary et al. 2020).

The contrasting trends between districts underscore the importance of localized strategies. Except for taro, the conservation status of the target crops seems far more of a concern in Mai Son than in Sa Pa. This is also confirmed by three pumpkin varieties already reported as lost in some of the focus groups in Mai Son, while, in the case of Sa Pa, only one variety of taro was reported as lost. Further investigation is required to understand whether the higher risk of loss in Mai Son is possibly related to the stronger commercial orientation of farmers, as suggested by Chaudhary et al. (2020) who indicate that traditional agricultural systems are under significant threat from modernization, market pressures, and environmental challenges.

The data and findings from this research can be used for multiple integrated conservation actions involving varietal diversity. First, as a baseline for future monitoring and timeline comparisons. Second, to prioritize in situ conservation interventions which could range from biodiversity seed fairs, cataloging, rematriation, community seed banks to cultural reaffirmation though education (de Haan et al. 2021). Third, to incorporate threatened farmer varieties into ex situ or genebank collections. Furthermore, the results of this study can inform additional studies to deepen our understanding of the structure and conservation status of variety populations. This could involve genetic analysis of in situ populations and their comparison with ex situ collections from the same regions in the past. The 5-cell methods, while offering a robust participatory approach, cannot distinguish genetic differences among farmer varieties with synonyms or homonyms. Especially in a multilingual context such as represented by northwestern Vietnam.

## Conclusion

The findings of this study underscore the critical importance of thoroughly assessing agrobiodiversity and documenting the associated traditional knowledge within farming systems in order to devise effective strategies to conserve valuable genetic resources for future generations. Some recommendations can be drawn from our study.

First, the importance of ethnobotanical knowledge and the people who hold it have been largely underestimated (Ragavan 2001; Dulloo et al. 2017). For millennia, word of mouth has served as the primary method for transmitting traditional knowledge across generations. This is knowledge that is also vulnerable to loss. Clearly, local expert knowledge and opinion about vernacular names and the conservation status of specific varieties has substantially informed this study. The documentation of traditional knowledge combined with a deeper understanding of the relationship between the knowledge, local people’s culture, and diverse resources is crucial to the development of the conservation strategies (Sheng-Ji 2001). Additional research should be conducted for documenting the rich ethnobotanical knowledge that the communities in the mountainous areas of Vietnam have shown to possess.

Second, the disparities in varietal diversity and conservation status across ethnic groups and districts provide a vital baseline for understanding the conservation dynamics of agrobiodiversity in Northwest Vietnam. This baseline should serve as a foundation for monitoring changes in agrobiodiversity over time and space, enabling the development of targeted conservation strategies in the face of the rapid agricultural transformation that the country is now undergoing. Some of these potential strategies have been highlighted in the discussion section.

Third, our results highlight the need for community-specific interventions. Strengthening the capacities of ethnic groups to maintain and utilize diverse varieties can enhance local food security, resilience, and cultural heritage. Strategies should respond to the specific challenges they aim to address. For instance, Padulosi and Dulloo (2012) suggest that rare and endangered varieties (i.e., grown by few farmers on a small area), besides being conserved in the national gene bank (ex situ conservation), could be supported through the establishment of community seed bank, rematriation programs, or the recognition and provision of incentives for custodian farmers (in situ conservation). On the other hand, options for the conservation of varieties grown by many farmers on a small area include promoting their continued use through seed-saving programs, identification and targeting of niche markets, or education of the communities on their dietary benefits to increase their consumption.

Finally, the 5-cell method employed in this study demonstrates its utility as a participatory approach for assessing agrobiodiversity. Expanding its application can help track trends in varietal diversity and inform adaptive management strategies. Future research should explore the use of additional methods (e.g., genetic analysis), socioeconomic drivers of varietal loss in relation to age and gender, and evaluate the impact of distinct conservation interventions on genetic diversity.

## Supplementary Information

Below is the link to the electronic supplementary material.

Supplementary file1 (PDF 775 KB)

## Data Availability

The dataset generated by the survey research and analyzed for this study is available in the Dataverse repository, 10.21223/6EKR4X.

## Footnotes

1 Defined cutoff points varied across crops and villages. Cutoff points for few vs many farmers ranged between 20 and 80%. Cutoff points for small vs large areas showed extreme variability and were defined by number of plants for banana and pumpkin; by number of plants or sqm for taro; and by number of raised beds for mustard green.

2 Traditionally, the soft core of the trunk of the banana variety *Co nguồn* grown by the Thai people was used to make an indispensable dish served at wedding ceremonies. However, the massive demand for this banana trunk at a specific time greatly impacted the natural growth and maintenance of this variety. According to respondents, the flowers and fruits of this variety can hardly be seen due to the harvesting of the trunk at the vegetative stage.
