# Supplementary material for: Varietal diversity and conservation status of banana, taro, pumpkin, and mustard green in mountainous areas of Northwest Vietnam
Source: Ambio. 2025 Oct 23;55(4):948–61. doi: 10.1007/s13280-025-02262-1 (PMC12960289; doi:10.1007/s13280-025-02262-1)
Supplement: Supplementary file 1 — Supplementary file1 (PDF 775 KB) [file 13280_2025_2262_MOESM1_ESM.pdf]

Supplementary Information

Article title: Varietal diversity and conservation status of banana, taro, pumpkin and mustard green in mountainous areas of Northwest Vietnam

Journal name: *Ambio*

Authors: Dang Toan Vu, Phuong Diep Vien Ta, Tuong Dang Vu, Diego Naziri, Le Thi Minh Thao, Vo Anh Thu, Israel Navarrete, Stef de Haan

Corresponding author:

Diego Naziri
International Potato Center, Hanoi, Vietnam
Natural Resources Institute (NRI), University of Greenwich, Chatham Maritime, UK
Email: d.naziri@greenwich.ac.uk

Appendix S1. Location of study sites. On the left, a map showing the target districts in Son La (Mai Son) and the target communes within the district. On the right, a map showing the target district in Lao Cai (Sa Pa) and the target communes within the district *(credit to Le Vinh Bui)*.

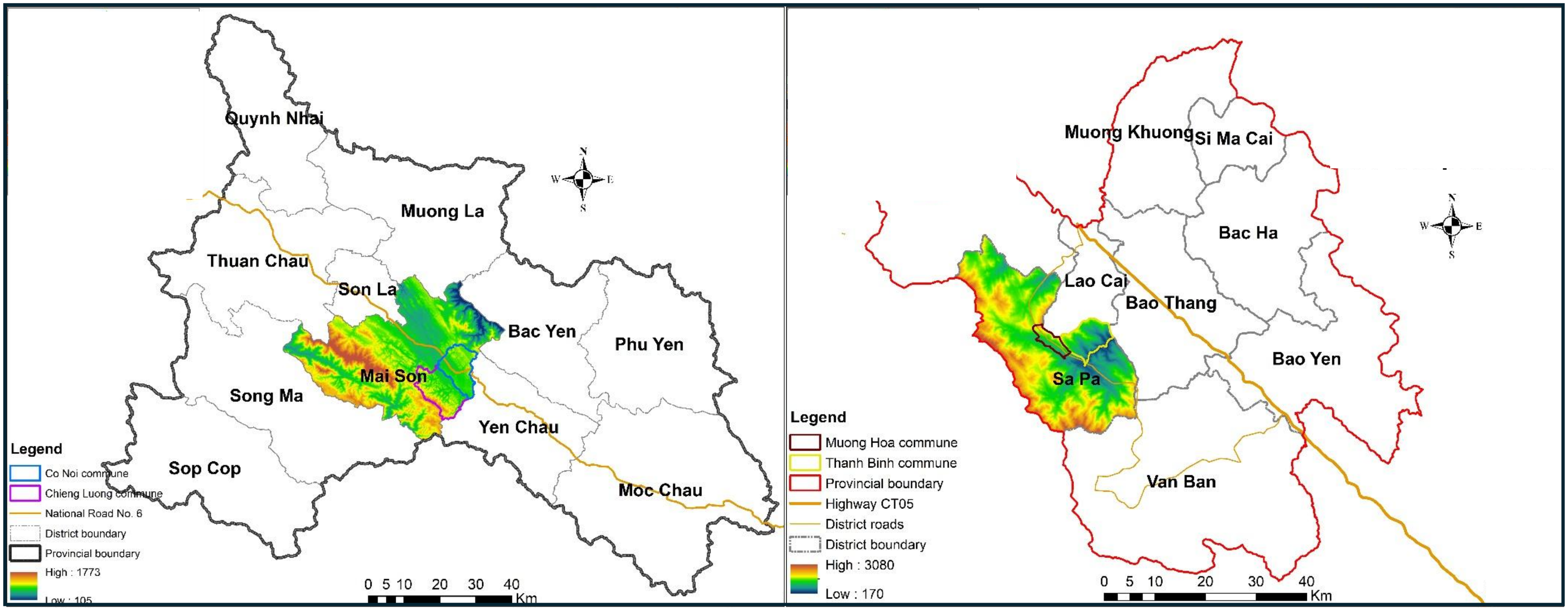

Appendix S2. Examples of farmer varieties identified in the survey. a: Cuoi Kia (Buddha hand banana) from Mai Son, Mac Phua Tim (Purple taro) from Mai Son, c: Phan nhum chuy (Pestle-shaped pumpkin) from Sa Pa, d: Ron ram pac (Hairy white Hmong mustard green) from Sa Pa. Extended photographic documentation and morphological characterization of identified farmer varieties in Sa Pa and Mai Son can be found at Vu et al. (2024b) and Vu et al. (2024c), respectively.

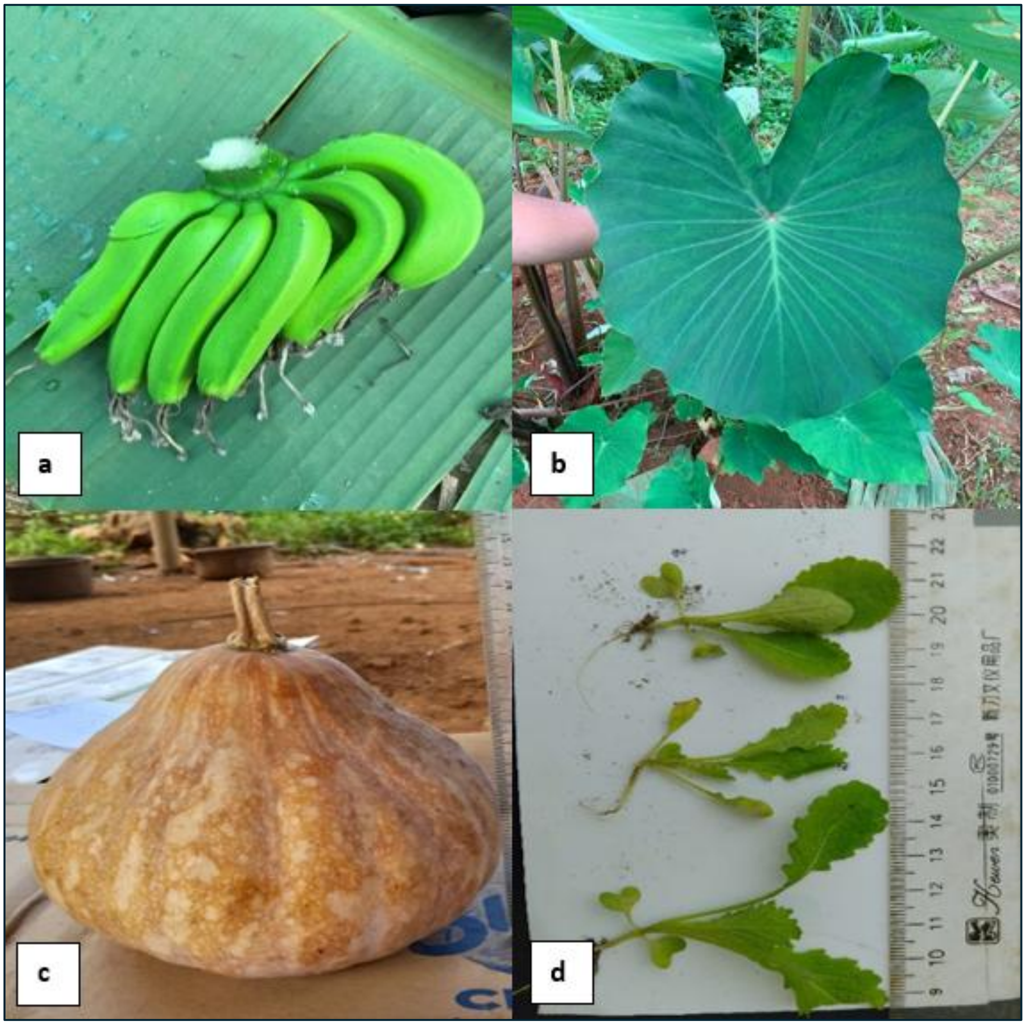

Appendix S3. Number of varieties reported by crop and district.

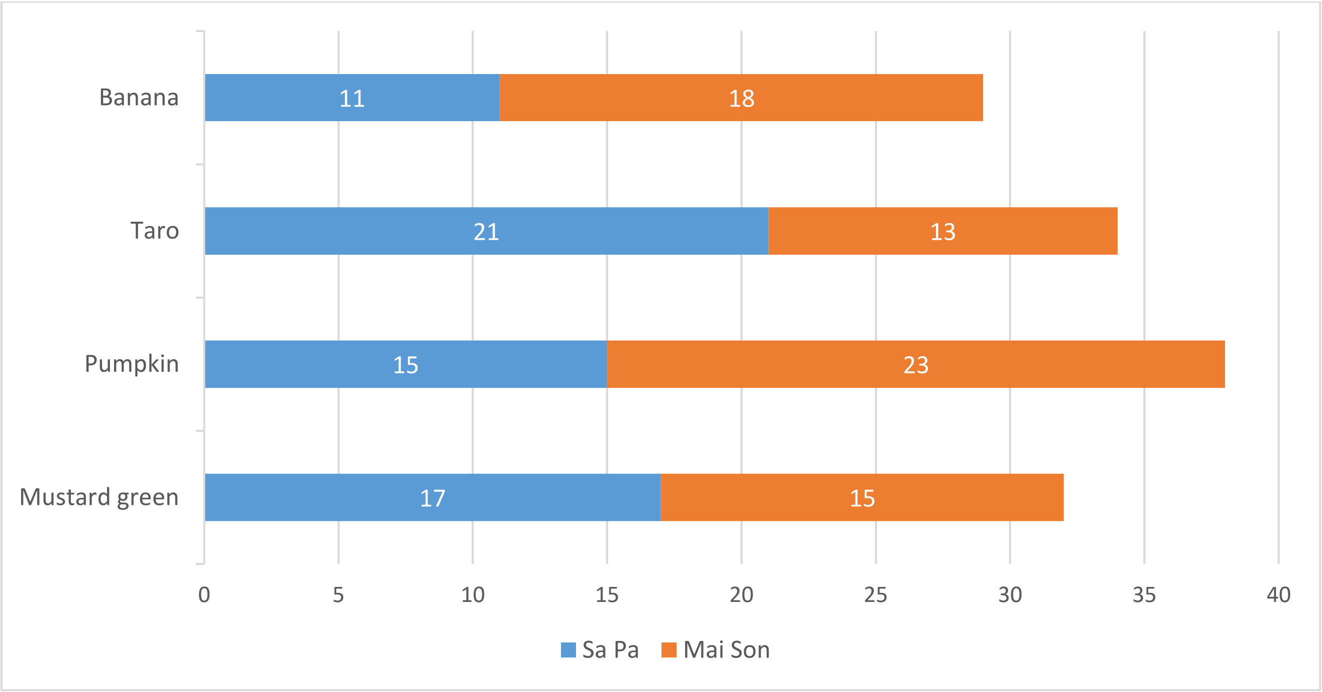

Appendix S4. Ethnobotanical uses of banana, taro, pumpkin, and mustard green varieties in Sa Pa and Mai Son districts of Vietnam

No.	Vernacular name (Ethnicity)	Name meaning	Features noted by respondents	Part used	Preparation	Use
**BANANA**						
1	Trơ trừ (Hmong)	"Trơ" means banana, "trừ" means sweet	The fruit is a-span-long and has a sweet flavor. The peel has slight ridges. It is intercropped with "Cắng dử".	Leaf, Trunk	Chopped, cooked	Fodder
				Fruit	Fresh	Consumption, sale (if surplus)
				Leaf	Dried	Wrapping cake
2	Trơ Mèo / Trơ cau (Hmong)	"Trơ" means banana, "Mèo" means Hmong, "cau" means sour flavor	The plant is tall, and the bunch is long. The fruit is big, nearly two-spans long, fragrant but it has a sour flavor. It requires large planting distances if not it is not going to produce fruits.	Flower, Fruit	Fresh	Culinary, sale (if surplus)
				Trunk, Flower	Juice	Diarrhea
3	Noọng chìu / Chìu me (Dao)	"Noọng chìu" means banana in general	The plant is tall and distributed in wet valleys and stream banks. The pseudostem is brownish yellow. The bunch is big and the fruit has many seeds inside.	Leaf	Fresh or dried	Wrapping cake
				Leaf	Fresh	Sale
				Trunk	Chopped, cooked	Fodder
				Trunk	Juice	Hangover cures, detoxification
4	Chìu piếu pẻ (Dao)		The fruit is small but fragrant. The peel is thin.	Flower	Fresh	Culinary
				Trunk	Chopped, cooked	Fodder (constipation treatment for pig)
				Fruit	Fresh	Consumption, sale
5	Chìu piếu ai (Dao)		The plant is very short.	Fruit	Fresh	Consumption, sale
				Trunk	Chopped, cooked	Fodder
6	Chìu piếu suy (Dao)		The fruit is big, long, and has a sour flavor. The peel is thick and has ridges.	Flower, Fruit	Fresh	Culinary
				Fruit	Fresh	Sale
				Trunk	Chopped, cooked	Fodder
7	Nòm chìu xi (Dao)	"Nòm" means leaf, "chìu xi" means red flower	The flower is red. The fruit is small green. The number of fruits per hand is low. The pseudostem is dark green. (The variety was taken from the local forest and was planted in the home garden.)	Flower, Trunk (soft core)	Cooked	Culinary
				Flower	Fresh	Sale
				Flower	Grilled	Shortness of breath
				Trunk	Chopped, decoction for bathwater	Health improvement for postpartum and preterm birth women

No.	Vernacular name (Ethnicity)	Name meaning	Features noted by respondents	Part used	Preparation	Use
8	Chìu cháng (Dao)		The fruit has many seeds, fragrant, and astringent taste. (The variety was taken from the local forest and was planted in the home garden.)	Trunk	Chopped, cooked	Fodder
9	Chìu piếu đang (Dao)		The plant is tall. At the young stage, the pseudostem is purple, when the plant grows, it turns green.	Fruit	Fresh	Consumption, sale
				Trunk	Chopped, cooked	Fodder
10	Nòm chìu piếu á (Dao)		The plant is quite short. The fruit is quite small.	Trunk	Chopped, cooked	Fodder
11	Nòm chìu chảng (Dao)		The plant is short and small. The fruit has a sour taste. (The variety was taken from the local forest and was planted in the home garden).	Trunk	Chopped, cooked	Fodder
12	Chuối tiêu (Kinh) / Cò mặc cuối tin tặp (Thai)	"Cò" means plant, "mặc cuổi" means banana, "tin tặp" means short	The plant is short. The leaf is long, oval, and dark green. The fruit is long, curved, fragrant, and has a sweet taste.	Fruit	Fresh	Consumption, sale
				Fruit	Sliced, Dried, Milled, Rounded off	Abdominal pains
				Trunk, Flower	Chopped, cooked	Fodder
				Leaf	Dried	Wrapping cake
				Leaf	Decoction	Diarrhea in pigs (Colibacillosis)
13	Chuối tây / Chuối sứ (Kinh) / Cò mặc cuổi kéo (Thái) Co cuổi xáy (Thái)	"Cò" means plant, "mặc cuổi" means banana, "kéo" means tall, "xáy" means egg-shaped	The plant is fastidious. It is susceptible to pseudostem borer.	Fruit, Flower, Trunk (soft core)	Fresh	Culinary, sale
				Leaf	Dried	Wrapping cake
				Trunk, Leaf	Chopped, cooked	Fodder
				Fruit	Ripe, Sliced, Dried	Back pains
				Fruit	Unripe/Ripe, Sliced, Dried, Sugar-soaked	Crunchy/Gummy sugar-coated candy
				Leaf petiole	Dried	Cordage
				Fruit	Fresh, Sliced, sugar-soaked	Stroke prevention
				Whole plant	Fresh	Sale
14	Chuối lá (Kinh) / Cò mặc cuổi xộp (Thái) / Mạ cuổi luông thòn (Thái)	"Cò" means plant, "mặc cuổi" means banana, "xộp" means sour flavor	The plant is tall. The pseudostem is big. The fruit has a sour flavor.	Fruit, Flower	Fresh	Culinary
				Trunk, leaf	Chopped, cooked	Fodder
15	Chuối ngự (Kinh)		The fruit is small and short but has fragrant and sweet taste. The pseudostem is susceptible to stem borer.	Fruit	Fresh	Consumption
				Trunk	Chopped, cooked	Fodder

No.	Vernacular name (Ethnicity)	Name meaning	Features noted by respondents	Part used	Preparation	Use
16	Cuổi pá 1 (Thai)	"Cuổi" means banana, "pá" means forest	The plant is distributed in dry lands. The fruit has many seeds inside. (The variety was taken from the local forest and was planted in the home garden.)	Trunk	Chopped, cooked	Fodder
				Fruit	Dried, Alcohol-soaked	Kidney stone
17	Cuổi pá 2 (Thai) / Cuổi nguộc (Thai)	"Cuổi" means banana, "pá" means forest, "nguộc" means smooth leaf	The plant is distributed in wet valleys near streams and rivers. It is tall, and the pseudostem is huge looked like a pillar. (The variety was taken from the local forest and was planted in the home garden.)	Young stem, young leaf, flower	Fresh	Culinary
18	Co nguồn (Thai)		The plant base is big, grows alone.	Trunk (soft core)	Cooked	Culinary (traditional dish)
				Flower	Cooked	Culinary
				Trunk, Leaf	Cooked	Fodder
				Fruit	Alcohol-soaked	Kidney stone
19	Cuổi buốp (Thai)		The fruit is big and sweet. The peel has ridges. Highly susceptible to pests and diseases.	Fruit, Flower	Fresh	Culinary
				Trunk	Chopped, cooked	Fodder
20	Cuổi bi khôm/ Mạ cuổi bi khôm (Thai)		Similar to No. 12 but the plant is taller and the fruit is smaller, shorter, and sourer.	Fruit	Fresh	Consumption, Sale
21	Cuổi cáy/ Mạ cuổi cáy/ Cuổi cáy nọi (Thai)		The fruit is small. The fruit flesh is little.	Fruit, Flower	Fresh	Culinary
				Trunk	Chopped, cooked	Fodder
22	Co cuổi quai (Thai)	"Co" means plant, "cuổi" means banana, "quai" means buffalo	The fruit is big, very short, has black seeds, and sweet taste.	Fruit, Flower	Fresh	Culinary
				Trunk	Chopped, cooked	Fodder
				Whole plant	Fresh	Sale
23	Mạ cuổi keo (Thai)	"Mạ" means fruit	The plant is taller than an adult person.	Fruit	Fresh	Consumption, Sale
				Leaf	Dried	Wrapping cake
24	Co cuổi típ/ Cuổi típ (Thai)		Similar to No.22, the plant is big and tall. But, the fruit is smaller than of No.22, has black seeds, and overly sweet taste. Highly susceptible to pests and diseases.	Fruit	Fresh	Consumption
				Trunk	Chopped, cooked	Fodder
25	Cuổi báu mi ma toòng (Thai)			Fruit	Fresh	Consumption
				Trunk	Chopped, cooked	Fodder
26	Cuổi mư nang (Thai)	"Cuổi" means banana, "mư" means hand, "nang" means fairy	Similar to No. 12 but the plant is taller. The bunch is small and has few hands. The fruit has curved shape like fairy's hand. The fruit has fragrant and sweet taste.	Fruit	Fresh	Consumption
				Trunk	Chopped, cooked	Fodder

No.	Vernacular name (Ethnicity)	Name meaning	Features noted by respondents	Part used	Preparation	Use
27	Cuổi mi kén (Thai)		The pseudostem is big. The fruit has many seeds.	Fruit	Sliced, Dried, Alcohol-soaked	Kidney tonic
				Trunk	Chopped, cooked	Fodder
28	Cuổi kia (Thai)	"Cuổi" means banana, "kia" means bat	The plant is small and short. The maximum pseudostem diameter is about 15cm which is not a preferred characteristic. The fruit is small, fragrant, and has a sweet taste.	Fruit	Fresh	Consumption
				Trunk	Chopped, cooked	Fodder
29	Cuổi Lào (Thai)	"Cuổi" means banana, "Lào" means Laos country	The plant is tall. The pseudostem is purple. The bunch is long with an average weight of 25kg. The fruit is short. Variety introduced from Laos.	Fruit	Fresh	Consumption, Sale
**TARO**						
1	Cắng dử (Hmong)		Prefers dry-land cultivation, requires small land area to develop, root widespread quickly.	Petiole	Cooked	Culinary
				Petiole	Cooked with chicken	Health improvement for postpartum women and skinny person
2	Cò đìa (Hmong)	"Cò" means taro	Prefers wetlands, normally distributed along the ditch.	Corm, Petiole, Stolon	Cooked	Culinary
				Whole plant	Cooked	Fodder
				Stolon	Fresh	Sale
3	Cò cay 1 / Cò cay còn trăng (Hmong)	"Cò" means taro, "cay" means oval, "còn trăng" means purple	Intercropping with maize in highlands, good flavor.	Corm, Petiole	Cooked	Culinary
				Corm, Petiole	Fresh	Sale
				Whole plant	Cooked	Fodder
4	Cò cài trơ / Cò kí thờ (Hmong)		Prefers dry-land, big corm but small numbers of corm, low yield, plant can cause itchy feeling.	Corm	Cooked	Culinary
5	Cò cay 2 / Cò cay đơng (Hmong)	"Cò" means taro, "cay" means oval, "đơng" means white		Corm	Cooked	Culinary
				Corm	Fresh	Sale
				Leaf, Petiole	Cooked	Fodder
6	Cò chua (Hmong)		Big stem, big leaf, big corm, plant can cause itchy feeling.	Corm	Cooked	Culinary
				Leaf, Petiole	Cooked	Fodder
				Corm	Fresh	Sale
7	Cò trơ (Hmong)	"Cò" means taro, "trơ" means banana	Long and big toe-size corm.	Corm	Cooked	Culinary
				Leaf, Petiole	Cooked	Fodder
8	Bon / Mon (Dao)	"Bon" means taro	Prefers wetlands, green petiole.	Petiole, Stolon	Cooked	Culinary
				Stolon	Fresh	Sale
				Leaf	Cooked	Fodder
9	Hậu (Dao)	"Hậu" means taro	Prefers dry-land cultivation, light brown petiole.	Corm, Petiole	Cooked	Culinary
				Corm	Fresh	Sale
10	Cờng hậu xi (Dao)		Cultivated in highlands, purple petiole.	Leaf, Petiole	Cooked	Culinary, fodder
				Petiole	Fresh	Sale

No.	Vernacular name (Ethnicity)	Name meaning	Features noted by respondents	Part used	Preparation	Use
11	Cờng hậu pẻ (Dao)		Prefers dry-land cultivation, greenish white petiole	Leaf, Petiole	Cooked	Culinary, fodder
				Petiole	Fresh	Sale
12	Hậu hau 1 (Dao)	"Hậu" means taro	Green petiole, long corm with a flavor which is not particularly appreciated by most consumers.	Corm	Cooked	Culinary
				Corm	Fresh	Sale
				Leaf	Cooked	Fodder
13	Hậu hau 2 (Dao)	"Hậu" means taro	Purple petiole, long corm with a flavor which is not particularly appreciated by most consumers.	Corm	Cooked	Culinary
				Corm	Fresh	Sale
14	Hậu đang (Dao)	"Hậu" means taro, "đang" means fragrant	Green leaf and petiole, round corm, good flavor.	Corm	Cooked	Culinary
				Corm	Fresh	Sale
				Leaf	Cooked	Fodder
15	Hậu xiền 1 (Dao)	"Hậu" means taro, "xiền" means red	Green leaf, purple petiole and corm, plant can cause itchy feeling, bad flavor.	Corm	Cooked	Culinary
				Corm	Fresh	Sale
				Leaf, Petiole	Cooked	Fodder
16	Hậu hau 3 / Chù nhiều hậu (Dao)	"Hậu" means taro	Light brown petiole, long corm, brown corm flesh.	Corm	Cooked	Culinary
				Corm	Fresh	Sale
				Leaf	Cooked	Fodder
17	Cờng hậu pưa (Dao)	"pưa" means big	Green petiole, bad flavor.	Petiole	Cooked	Culinary
				Petiole	Fresh	Sale
18	Hậu xiền 2 (Dao)	"Hậu" means taro, "xiền" means red	White purple petiole, white corm flesh with bad flavor.	Corm	Cooked	Culinary
				Corm	Fresh	Sale
				Leaf, Petiole	Cooked	Fodder
19	Hậu pưa (Dao)		Long white corm, green petiole, white petiole basal-ring, bad flavor.	Corm	Cooked	Culinary
				Leaf, Petiole	Cooked	Fodder
20	Hậu đàng xuý (Dao)		Purple petiole, big long corm, purple corm flesh, good flavor, not popular.	Corm	Cooked	Culinary
				Corm	Fresh	Sale
				Leaf, Petiole	Cooked	Fodder
21	Hờ hậu nháu (Dao)		Dark green petiole and leaf.	Corm	Crushed	Treating boils, leg pains
				Corm	Fresh	Sale
				Corm	Cooked	Yeast production
22	Khoai sọ dọc trắng (Kinh)		Small round corm, good flavor.	Corm	Cooked	Culinary
				Leaf, Petiole	Cooked	Fodder
23	Khoai sọ dọc tím (Kinh)		Big oval corm, tall plant, big leaf, plant can cause itchy feeling.	Corm	Cooked	Culinary
				Leaf, Petiole	Cooked	Fodder
24	Khoai sọ Thuận Châu (Kinh)		Round corm, very good flavor.	Corm	Cooked	Culinary
				Leaf, Petiole	Cooked	Fodder
25	Rau bon (Kinh) / Co bon ban (Thai)		Cultivated in wetlands, purple petiole junction, plant is higher than a person, round leaf.	Leaf, Petiole, Stolon	Cooked	Culinary
				Petiole, Stolon	Fresh	Sale
26	Rau bon ngứa (Kinh) / Co bon căn (Thai)	"căn" means non-edible	Absent petiole junction color, leaf is longer than Rau bon's, plant can cause itchy feeling, common in pond banks.	Whole plant	Cooked	Fodder

No.	Vernacular name (Ethnicity)	Name meaning	Features noted by respondents	Part used	Preparation	Use
27	Dọc mùng xanh (Kinh) / Phác sọ oóc (Thai)		Cultivated in highlands, light green to green petiole, well grow in paddy field, dying when intercroppped with maize in highlands due to the effect of maize's pesticide application.	Corm, Leaf, Petiole	Cooked	Culinary
				Petiole	Fresh	Culinary, Sale
				Leaf, Petiole	Cooked	Fodder
28	Dọc mùng tím (Kinh)		Purple petiole, rare.	Petiole	Cooked	Culinary
				Petiole	Fresh	Sale
29	Mạc phược hóm 1 / Mạ phựa hom (Thai)	"Mạc phược" or "Mạ phựa" means taro, "hom" means fragrant	Green petiole, round corm, fragrant, good flavor, being a fastidious variety, low yield and low productivity.	Corm	Cooked	Culinary
				Leaf, Petiole	Cooked	Fodder
30	Mạc phược hóm 2	"Mạc phược" means taro	Purple petiole, round corm, non-fragrant, bad flavor.	Corm	Cooked	Culinary
31	Mạc phược tìn / Mạ phựa lẳng (Thai)	"Mạc phược" or "Mạ phựa" means taro, "lẳng" means purple	Purple leaf main vein, petiole and corm, long and big corm, good flavor, little fragrance, non-itchy.	Corm	Cooked	Culinary
				Corm, Leaf, Petiole	Cooked	Fodder
32	Mạ phựa căn (Thai)	"Mạ phựa" means taro, "căn" means non-fragrant	Dark purple petiole, high plant, plant can cause itchy feeling, oval corm, in dry season only root part remains, leaf only appears in rainy season.	Corm	Cooked	Culinary
				Whole plant	Cooked	Fodder
33	Mạ phựa tím (Thai)		Round purple corm, plant can cause itchy feeling, bad flavor.	Whole plant	Cooked	Culinary, fodder
34	Bon mu (Thai)	"mu" means pig	Big dark green leaf, dark green petiole, white corm flesh, plant grows quickly.	Leaf, Petiole	Cooked	Fodder
**PUMPKIN**						
1	Tau 1 (Hmong)	"Tau" means pumpkin in general.	The fruit is pyriform and can be stored for consumption for a long period. Farmers can easily preserve the seeds for the next cropping season.	Fruit, Leaf, Young shoot	Cooked	Culinary
				Whole plant (except root)	Cooked	Fodder
2	Tau 2 (Hmong)	"Tau" means pumpkin in general.	The fruit is small and in dumbbell shape. Farmers face difficulty in keeping the seeds for later use because this variety is prone to be hybridized with other varieties. Although this variety is cultivated in a small area, the productivity still meets the farmer's demand for family consumption.	Fruit	Cooked	Culinary
				Whole plant (except root)	Cooked	Fodder
3	Tau 3 (Hmong)	"Tau" means pumpkin in general.	The fruit is bigger than "Tau 2", flattened with deep ribs on the cortex. Farmers can easily preserve the seeds for the next cropping season.	Leaf, Young shoot	Cooked	Culinary
				Whole plant (except root)	Cooked	Fodder
4	Tau 4 (Hmong)	"Tau" means pumpkin in general.		Fruit, Leaf, Young shoot	Cooked	Culinary

No.	Vernacular name (Ethnicity)	Name meaning	Features noted by respondents	Part used	Preparation	Use
			The fruit is pyriform. Fruit flesh is orange, with a fragrant and sweet flavor when it is cooked.	Whole plant (except root)	Cooked	Fodder
5	Tau cháng đầu (Hmong)	"Tau" means pumpkin in general.	Farmers face difficulty in keeping the seeds for later use because this variety is prone to be hybridized with other varieties.	Fruit, Leaf, Young shoot	Cooked	Culinary
				Whole plant (except root)	Cooked	Fodder
6	Phản nhum chuỳ (Dao)	"Phản nhum" means pumpkin in general, "chùy" means thigh (the fruit as big as a thigh)	The fruit is cylindrical.	Fruit, Flower, Leaf, Young shoot, Seed	Cooked	Culinary
				Leaf, Stem	Cooked	Fodder
				Seed	Dehusked, Crushed, Mixed with sugar	Anthelmintic drug
				Fruit	Fresh	Sale
7	Phản nhum đao (Dao)	"Phản nhum" means pumpkin in general, "đao" means long	The fruit is in an elongated form. Due to the fewer number of seeds and the difficulties in cultivation, this variety is difficult to propagate.	Fruit, Flower, Leaf, Young shoot, Seed	Cooked	Culinary
				Leaf, Stem	Cooked	Fodder
				Seed	Dehusked, Crushed, Mixed with sugar	Anthelmintic drug
				Fruit	Fresh	Sale
8	Phản nhum chùn (Dao)	"Phản nhum" means pumpkin in general, "chùy" means round shape	The fruit is globular with many seeds leading to the propagation of farmers with great ease.	Fruit, Flower, Leaf, Young shoot, Seed	Cooked	Culinary
				Leaf, Stem	Cooked	Fodder
				Seed	Dehusked, Crushed, Mixed with sugar	Anthelmintic drug
				Fruit	Fresh	Sale
9	Phản nhum bụt (Dao)	"Phản nhum" means pumpkin in general	The fruit is pyriform. Fruit flesh is orange, with a fragrant and sweet flavor when it is cooked. However, the fruit does not last long and contains few seeds, which makes propagation in the next season difficult.	Fruit, Flower, Leaf, Young shoot, Seed	Cooked	Culinary
				Leaf, Stem	Cooked	Fodder
				Seed	Dehusked, Crushed, Mixed with sugar	Anthelmintic drug
				Fruit	Fresh	Sale
10	Phản nhum pìn (Dao)	"Phản nhum" means pumpkin in general, "pin" means wheel	The fruit is flattened. The stem-end and the bottom-end of the fruit are depressed. The fruit has many seeds, which makes the propagation of farmers easier.	Fruit, Flower, Leaf, Young shoot, Seed	Cooked	Culinary
				Leaf, Stem	Cooked	Fodder
				Seed	Dehusked, Crushed, Mixed with sugar	Anthelmintic drug
				Fruit	Fresh	Sale
11	Phản nhung đáo (Dao)	"Phản nhung" means pumpkin in general	The fruit is cylindrical. This variety is normally cultivated in the home garden, where it climbs on the rooftop, so it does not take up much space.	Fruit	Cooked	Culinary
				Seed	Dehusked, Crushed, Mixed with sugar	Anthelmintic drug
12				Fruit	Cooked	Culinary

No.	Vernacular name (Ethnicity)	Name meaning	Features noted by respondents	Part used	Preparation	Use
	Phản nhung chuỳ (Dao)	"Phản nhung" means pumpkin in general	The fruit is dumbbell-shaped. This variety is normally cultivated in the home garden, where it climbs on the rooftop, so it does not take up much space.	Seed	Dehusked, Crushed, Mixed with sugar	Anthelmintic drug
13	Phản nhung chùn (Dao)	"Phản nhung" means pumpkin in general	The fruit is globular. This variety is cultivated in maize fields.	Leaf, Young shoot	Cooked	Culinary
				Whole plant (except root)	Cooked	Fodder
14	Phản nhung ping (Dao)	"Phản nhung" means pumpkin in general	The fruit is flattened. This variety is cultivated in maize fields.	Leaf, Young shoot	Cooked	Culinary
				Whole plant (except root)	Cooked	Fodder
15	Phản nhung bụt (Dao)	"Phản nhung" means pumpkin in general	The fruit is pyriform. This variety is cultivated in maize fields.	Leaf, Young shoot	Cooked	Culinary
				Whole plant (except root)	Cooked	Fodder
16	Bí bánh xe 1 (Kinh)		The fruit is flattened with deep ribs. The fruit does not last long, but its variety is a preference of farmers due to its good flavor. So, it is easy to sell.	Fruit	Cooked	Culinary
				Fruit	Fresh	Sale
17	Bí bánh xe 2 (Kinh)		The fruit is flattened with superficial ribs. This fruit has a good flavor and is easy to sell.	Fruit	Cooked	Culinary
				Fruit	Fresh	Sale
18	Bí bầu dục (Kinh)		The fruit is oval and has a bad flavor.	Fruit	Cooked	Culinary
				Fruit	Fresh	Sale
19	Bí hồ lô (Kinh)		The fruit is pyriform has a bad flavor. This variety has low yield.	Fruit	Cooked	Culinary
				Fruit	Fresh	Sale
20	Bí nếp (Kinh)		The fruit is pyriform. The yield is low.	Fruit	Cooked	Culinary
				Fruit	Fresh	Sale
21	Bí múi (Kinh)		The fruit has a good flavor, but the fruit size is too big, so it does not suit farmer's preference. This variety is hybrid, so the farmers cannot keep the seeds for the next season leading to low cultivation efficiency.	Fruit	Cooked	Culinary
				Fruit	Cooked with green beans	Headache
				Fruit	Fresh	Sale
22	Mã ức môn 1 (Thai)	"Mã ức" means pumpkin in general	The farmers plant this variety for their own consumption.	Fruit, Young shoot	Cooked	Culinary
23	Mã ức môn 2 (Thai)	"Mã ức" means pumpkin in general		Fruit, Young shoot	Cooked	Culinary
24	Mã ức hi (Thai)	"Mã ức" means pumpkin in general	This variety is not preferred due to many difficulties in cultivation, few seeds, and low yield.	Fruit, Young shoot	Cooked	Culinary
25	Mã ức môn 3 (Thai)	"Mã ức" means pumpkin in general	The fruit size is too big (up to 20kg in weight), so it does not suit farmer's preference	Fruit, Young shoot	Cooked	Culinary

No.	Vernacular name (Ethnicity)	Name meaning	Features noted by respondents	Part used	Preparation	Use
26	Mã ức thuồn (Thai)	"Mã ức" means pumpkin in general		Fruit, Young shoot	Cooked	Culinary
27	Mã ự 1 (Thai)	"Mã ự" means pumpkin in general	The fruit is flattened with deep ribs.	Fruit, Leaf, Young shoot	Cooked	Culinary
28	Mã ự 2 (Thai)	"Mã ự" means pumpkin in general	The fruit is flattened with superficial ribs.	Fruit, Leaf, Young shoot	Cooked	Culinary
29	Mã ự 3 (Thai)	"Mã ự" means pumpkin in general	The fruit is flattened.	Fruit, Leaf, Young shoot	Cooked	Culinary
30	Mã ự 4 (Thai)	"Mã ự" means pumpkin in general	The fruit is big and flattened. This variety is intercropped with maize in the maize fields. The application of herbicides and pesticides to maize production seriously affects the growth of this variety, which leads to low yield.	Fruit, Leaf, Young shoot	Cooked	Culinary
				Whole plant (except root)	Cooked	Fodder
31	Mã ự hua chua (Thai)	"Mã ự" means pumpkin in general	The fruit is pyriform.	Fruit, Leaf, Young shoot	Cooked	Culinary
32	Mã ự xạ mong (Thai)	"Mã ự" means pumpkin in general	The fruit is elongated.	Fruit, Leaf, Young shoot	Cooked	Culinary
33	Mã ự 1 (Thai)	"Mã ự" means pumpkin in general	The fruit is flattened with deep ribs.	Fruit, Leaf, Young shoot	Cooked	Culinary
34	Mã ự 2 (Thai)	"Mã ự" means pumpkin in general	The fruit is flattened with superficial ribs.	Fruit, Leaf, Young shoot	Cooked	Culinary
35	Mã ự 3 (Thai)	"Mã ự" means pumpkin in general	The fruit is flattened.	Fruit, Leaf, Young shoot	Cooked	Culinary
36	Mã ự 4 (Thai)	"Mã ự" means pumpkin in general	The fruit is big and flattened. This variety is intercropped with maize in the maize fields. The application of herbicides and pesticides to maize production seriously affects the growth of this variety, which leads to low yield.	Fruit, Leaf, Young shoot	Cooked	Culinary
				Whole plant (except root)	Cooked	Fodder
37	Mã ự hua chua (Thai)	"Mã ự" means pumpkin in general	The fruit is pyriform.	Fruit, Leaf, Young shoot	Cooked	Culinary
38	Mã ự xạ mong (Thai)	"Mã ự" means pumpkin in general	The fruit is elongated.	Fruit, Leaf, Young shoot	Cooked	Culinary
**MUSTARD GREEN**						
1	Ron chua (Hmong)		Green leaf and petiole, long harvesting time. The plant has a bitter flavor.	Whole plant (except root)	Cooked	Culinary
				Whole plant	Fresh	Sale
2	Ron ron la (Hmong)		The plant grows quickly, but the harvesting time is short because of early flower development (normally 1 month earlier than "Ron chua"). The plant has a sweet flavor.	Whole plant (except root)	Cooked	Culinary
				Whole plant	Fresh	Sale

No.	Vernacular name (Ethnicity)	Name meaning	Features noted by respondents	Part used	Preparation	Use
3	Ron ron du (Hmong)		Flower bloom late (normally 1 month later than "Ron chua")	Whole plant (except root)	Cooked	Culinary
				Whole plant	Fresh	Sale
4	Ron răm pặc (Hmong)		Long whitish-green petiole, flower bloom very fast. The plant has a good flavor.	Whole plant (except root)	Cooked	Culinary
				Whole plant	Fresh	Sale
5	Ron đơ (Hmong)		Whitish-green petiole, leaf pubescence absent	Whole plant (except root)	Cooked	Culinary
				Young shoot	Fresh	Sale
6	Lay chải (Dao)	"Lay" means vegetable, "chái" means mustard	This is the main mustard variety in the household. The variety can be planted in winter and harvested after one month after planting.	Whole plant (except root)	Cooked	Culinary
				Whole plant	Fresh	Sale
				Whole plant	Fresh or cooked	Fodder
7	Lay bết (Dao)		The productivity from single bed can be enough for consumption of a family. This variety has a non-bitter flavor	Whole plant (except root)	Cooked	Culinary
				Whole plant	Fresh	Sale
				Whole plant	Fresh or cooked	Fodder
8	Lay hoán (Dao)		This variety has a cold-tolerant ability. The harvesting time is long, up to 3 months. The variety has a bitter flavor.	Whole plant (except root)	Cooked	Culinary
				Whole plant	Fresh	Sale
				Whole plant	Fresh or cooked	Fodder
9	Lay pẹ (Dao)	"Lay" means vegetable,	The harvesting time is short. The variety has a non-bitter flavor. Many households plant this variety due to high market demand.	Whole plant (except root)	Cooked	Culinary
				Whole plant	Fresh	Sale
				Whole plant	Fresh or cooked	Fodder
10	Lay chày meng (Dao)		This is the main mustard variety in the household. The planting time and harvesting time are short (about 1 month after planting). This variety has a sweet flavor but turns bitter as the plant ages.	Whole plant (except root)	Cooked	Culinary
				Whole plant	Fresh	Sale
11	Lay chày si (Dao)		The preference varies by the people's taste.	Whole plant (except root)	Cooked	Culinary
				Whole plant	Fresh or cooked	Fodder
12	Lay chày dom pe (Dao)		Due to bad flavor and long harvesting time, this variety does not attract farmer's preference.	Whole plant (except root)	Cooked	Culinary
13	Lay chày bết (Dao)		This variety can be harvested in a long period (more than 4 months) due to late flowering. Edible parts of this variety are soft and have a sweet flavor.	Whole plant (except root)	Cooked	Culinary
14	Lay chày dom chia (Dao)		The harvesting time is long. The variety has a bitter flavor.	Whole plant (except root)	Cooked	Culinary

No.	Vernacular name (Ethnicity)	Name meaning	Features noted by respondents	Part used	Preparation	Use
15	Lay chày heo 1 (Dao)		The harvesting time is long. The leaf surface is absent pubescence.	Whole plant (except root)	Cooked	Culinary
16	Lay chày heo 2 (Dao)		The harvesting time is long. The leaf surface is pubescence.	Whole plant (except root)	Cooked	Culinary
17	Lay chày pẹ (Dao)		Many households cultivate this variety. Cultivating faces difficulties. This variety has a bitter flavor.	Whole plant (except root)	Cooked	Culinary
18	Cải mèo 1 (Kinh)	"Cải mèo" means mustard green in general	Introduced since 1977 by farmers from Hoai Duc district, Hanoi.	Whole plant (except root)	Cooked	Culinary
				Whole plant	Fresh	Sale
19	Cải mèo 2 (Kinh)	"Cải mèo" means mustard green in general	Introduced since 1977 by farmers from Hoai Duc district, Hanoi.	Whole plant (except root)	Cooked	Culinary
				Whole plant	Fresh	Sale
20	Cải mèo 3 (Kinh)	"Cải mèo" means mustard green in general	Introduced since 1977 by farmers from Hoai Duc district, Hanoi.	Whole plant (except root)	Cooked	Culinary
				Whole plant	Fresh	Sale
21	Cải mèo 4 (Kinh)	"Cải mèo" means mustard green in general	Introduced since 1977 by farmers from Hoai Duc district, Hanoi.	Whole plant (except root)	Cooked	Culinary
				Whole plant	Fresh	Sale
22	Phác cát mẹo 1 (Thai)	"Phác cát" means mustard green	Many households cultivate this variety to serve their consumption, they only sell the plant if surplus.	Whole plant (except root)	Cooked	Culinary
				Whole plant	Fresh	Sale
23	Phác cát mẹo 2 (Thai)	"Phác cát" means mustard green	Many households cultivate this variety to serve their consumption, they only sell the plant if surplus.	Whole plant (except root)	Cooked	Culinary
				Whole plant	Fresh	Sale
24	Phác cát chằng (Thai)	"Phác cát" means mustard green	Cultivating faces difficulties due to the lack of seeds.	Whole plant (except root)	Cooked	Culinary
25	Phác cát soi (Thai)	"Phác cát" means mustard green	Many households cultivate this variety to serve their consumption, they only sell the plant if surplus.	Whole plant (except root)	Cooked	Culinary
				Whole plant	Fresh	Sale
26	Phác cát lậu (Thai)	"Phác cát" means mustard green	People have more choices with other varieties, so they don't prefer this one.	Whole plant (except root)	Cooked	Culinary
27	Phác cát mẹo 1 (Thai)		Many households cultivate this variety to serve their consumption, they only sell the plant if surplus.	Whole plant (except root)	Cooked	Culinary
				Whole plant	Fresh	Sale
28	Phác cát mẹo 2 (Thai)			Whole plant (except root)	Cooked	Culinary
29	Phác cát mẹo 3 (Thai)			Whole plant (except root)	Cooked	Culinary
				Whole plant	Fresh	Sale
30	Phác cát lón (Thai)			Whole plant (except root)	Cooked	Culinary

No.	Vernacular name (Ethnicity)	Name meaning	Features noted by respondents	Part used	Preparation	Use
31	Phác cát cản buống (Thai)			Whole plant (except root)	Cooked	Culinary
32	Phác cát mong (Thai)			Whole plant (except root)	Cooked	Culinary

Appendix S5. Varieties reported as being cultivated by few households on a small area, or already being lost, in all study villages in a district

	**Sa Pa**	**Mai Son**
**Banana**	**3 varieties**<br>Nòm chìu xi<br>Chìu cháng<br>Nòm chìu chảng	**6 varieties**<br>Chuối ngự<br>Cuổi nguộc / Cuổi pá<br>Cuổi buốp<br>Co cuổi típ / Cuổi típ<br>Cuổi kia<br>Cuổi Lào
**Mustard green**	**None**	**None**
**Pumpkin**	**None**	**10 varieties**<br>Bí bầu dục<br>Bí hồ lô<br>Bí nếp<br>Mã ức hi<br>Mã ức thuồn<br>Má ự 4<br>Má ự hua chua<br>Bí múi<br>Mã ức môn 3<br>Má ự xạ mong
**Taro**	**9 varieties**<br>Cò trơ<br>Cờng hậu xi<br>Hậu hau 1<br>Hậu hau 2<br>Hậu hau 3<br>Cờng hậu pưa<br>Hậu xiền 2<br>Hờ hậu nháu<br>Cò cài trơ	**6 varieties**<br>Khoai sọ dọc trắng<br>Khoai sọ dọc tím<br>Dọc mùng tím<br>Mạc phược hóm 2<br>Mạc phược tìn / Mạ phựa lẳng<br>Mạ phựa tím

Appendix S6. Summary distribution of varieties across the 5-cell quadrants by district

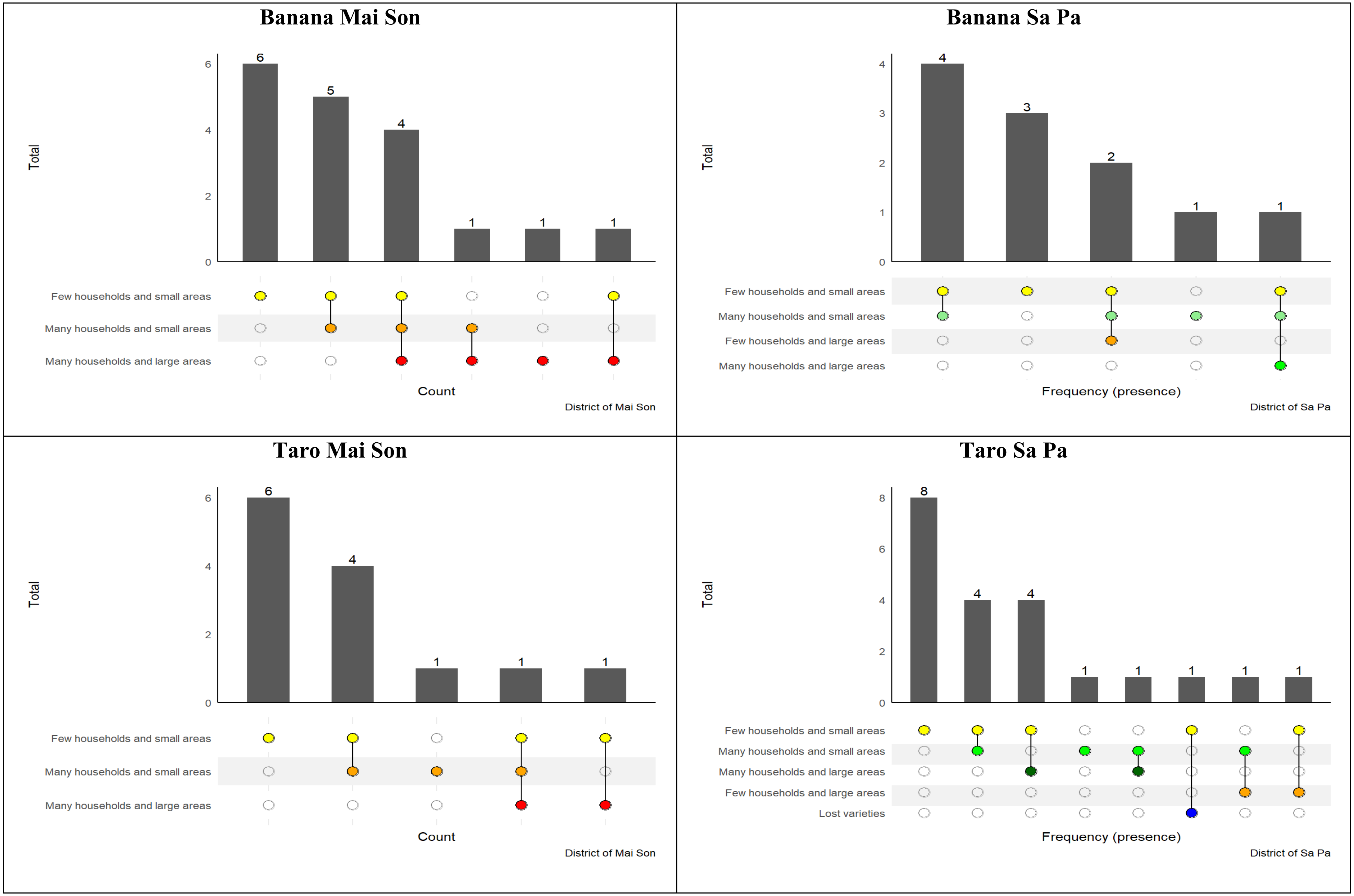

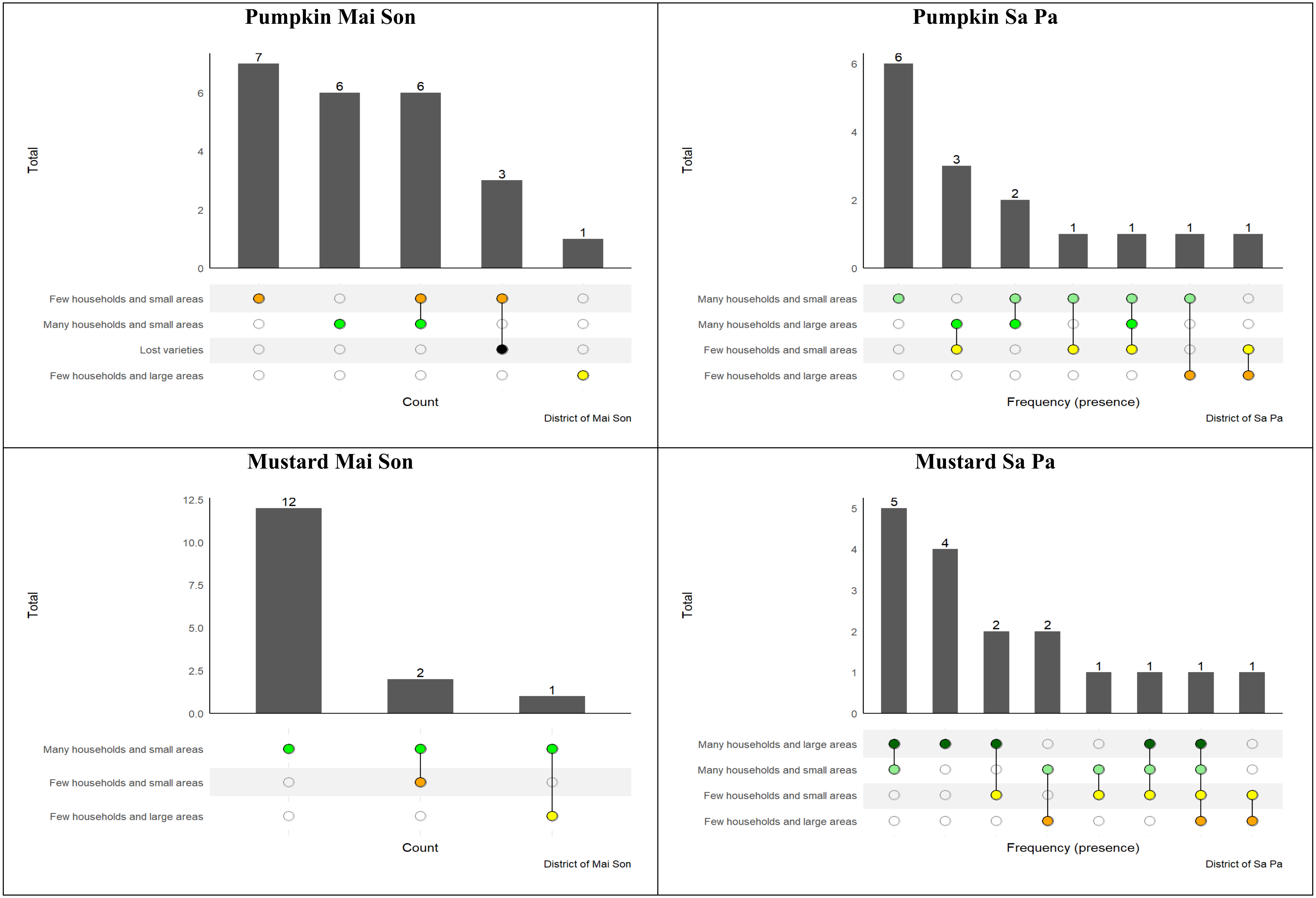

Pumpkin Mai Son
Total
7
6
6
3
1
Few households and small areas
Many households and small areas
Lost varieties
Few households and large areas
Count
District of Mai Son
Pumpkin Sa Pa
Total
6
3
2
1
1
1
1
Many households and small areas
Many households and large areas
Few households and small areas
Few households and large areas
Frequency (presence)
District of Sa Pa
Mustard Mai Son
Total
12
2
1
Many households and small areas
Few households and small areas
Few households and large areas
Count
District of Mai Son
Mustard Sa Pa
Total
5
4
2
2
1
1
1
1
Many households and large areas
Many households and small areas
Few households and small areas
Few households and large areas
Frequency (presence)
District of Sa Pa
